# Multiple Chronic Conditions and Limitations in Activities of Daily Living in a Community-Based Sample of Older Adults in New York City, 2009

**DOI:** 10.5888/pcd10.130159

**Published:** 2013-11-27

**Authors:** Nancy L. Ralph, Thelma J. Mielenz, Hilary Parton, Anne-Marie Flatley, Lorna E. Thorpe

**Affiliations:** Author Affiliations: Thelma J. Mielenz, Columbia University Mailman School of Public Health, New York, New York; Hilary Parton, New York City Department of Health and Mental Hygiene, Long Island City, New York; Anne-Marie Flatley, New York City Housing Authority, New York, New York; Lorna E. Thorpe, City University of New York School of Public Health at Hunter College, New York, New York.

## Abstract

**Introduction:**

Nationally, 60% to 75% of older adults have multiple (2 or more) chronic conditions (MCCs), and the burden is even higher among low-income, racial/ethnic minority populations. MCCs limit activities of daily living (ADLs), yet this association is not well characterized outside of clinical populations. We examined the association of MCCs with ADLs in a racially/ethnically diverse population of low-income older adults living in New York City public housing.

**Methods:**

A representative sample of 1,036 New York City Housing Authority residents aged 65 or older completed a telephone survey in June 2009. We examined the association of up to 5 chronic conditions with basic ADL (BADL) limitations, adjusting for potential confounders by using logistic regression.

**Results:**

Of respondents, 28.7% had at least 1 BADL limitation; 92.9% had at least 1 chronic condition, and 79.0% had MCCs. We observed a graded association between at least 1 BADL limitation and number of chronic conditions (using 0 or 1 condition as the reference group): adjusted odds ratio (AOR) for 3 conditions was 2.2 (95% confidence interval [CI], 1.3–3.9); AOR for 4 conditions, 4.3 (95% CI, 2.5–7.6); and AOR for 5 conditions, 9.2 (95% CI, 4.3–19.5).

**Conclusion:**

Prevalence of BADL limitations is high among low-income older adults and increases with number of chronic conditions. Initiating prevention of additional conditions and treating disease constellations earlier to decrease BADL limitations may improve aging outcomes in this population.

## Introduction

Between 60% and 75% of adults aged 65 or older have multiple chronic conditions (MCCs). This burden of MCCs is expected to grow as the large cohort born between 1946 and 1964 age and live longer and as the prevalence of certain chronic conditions increases in the population as a whole (1,2).

Many older adults experience limitations in their ability to perform basic activities of daily living (BADLs), such as bathing, eating, and getting in and out of a bed or chair. These limitations reduce an individual’s capacity for independent living and can predict nursing home admissions, frailty, and mortality (3). The risk of BADL limitations may increase with the presence of MCCs (3–5).

Both MCCs and BADL limitations are more common among people in low-income communities than those in higher-income communities (2,6), and racial/ethnic disparities are also well documented (7,8). For older adults with fewer resources, limited assistance with in-home management of complex medical conditions and options for alternative care facilities may put their overall health and well-being at greater risk (9).

Although the magnitude of association between specific chronic conditions and either loss of independence or difficulty performing certain functions may vary (10,11), limitations in performing BADL and instrumental ADLs (IADLs, or activities that include managing the household and community navigation, such as shopping, housework, and managing money) may be universal outcomes for a wide range of disease constellations (12).

Understanding both the distribution of MCCs and their potential effects on ADL limitations among low-income older adults is important in guiding targeted community interventions and planning to make housing and other facilities accommodations suitable to the aging population. Targeted medical interventions at the individual or subpopulation level can reduce health disparities in comorbidity and aging-associated disability. Yet, to date, the epidemiology of MCCs and their association with ADL limitations has not been well characterized in racially/ethnically diverse communities of low-income older adults (3). A limited body of literature suggests that the number of conditions — rather than the specific conditions, the severity of the conditions, or particular combinations of conditions — may be more closely associated with BADL and IADL limitations (12,13). The objective of this study was to examine the association between 5 chronic conditions and BADL and IADL limitations using data from the 2009 New York City Housing Authority (NYCHA) Senior Survey, a representative telephone survey of NYCHA older residents living in public housing (14). We hypothesized that a graded association would exist between the number of chronic conditions and the likelihood of BADL limitations.

## Methods

### Survey population and study design

NYCHA is the largest housing authority in the United States. With more than 400,000 residents, NYCHA manages subsidized housing for more than 61,500 low- and moderate-income adults aged 65 or older, most of whom are black or Hispanic. To assess a range of health outcomes and social conditions among NYCHA seniors, NYCHA, the New York City Department of Health and Mental Hygiene, the New York City Department for the Aging, and the City University of New York School of Public Health conducted a survey of residents aged 65 or older.

In June 2009, this partnership contracted with a vendor to conduct a cross-sectional, representative, systematic sample survey of older adults in NYCHA public housing. Interviews were conducted in English, Spanish, Russian, and Chinese with randomly selected older residents during June 2009. During a 3-week period, 1,036 telephone surveys collected data on numerous self-reported characteristics, including data on behaviors and physical and mental health diagnoses. The survey response rate was 34.7%, and the cooperation rate was 93.4%. Data were linked to same-year electronic administrative records in the NYCHA Tenant Data System to obtain demographic data, including age, race/ethnicity, income, disability status, and household makeup. Respondents were compared with nonrespondents on disability status (any, mobility, vision, mental, hearing, and wheelchair use) to confirm representativeness of the survey respondents. Only wheelchair use was significantly different (3% for respondents vs 5% for nonrespondents, *P* = .02). Details of this survey are available elsewhere (14). To ensure an accurate representation of the distribution of BADLs, participants missing valid responses (yes/no) for any of the 6 questions about BADLs (n = 20) were excluded from the study, resulting in a sample size of 1,016. Excluded participants did not differ from those in the study group by age, race/ethnicity, or income.

### Study variables

Our primary outcome of interest was BADL limitations, as measured by self-report of difficulty in 6 basic self-care activities: bathing, dressing, toileting, transferring (getting in and out of bed or chair), eating, and getting around the home (15). We grouped BADL limitations to describe 2 main outcomes by number of BADLs 1): at least 1 BADL limitation versus none and 2) 3 to 6 limitations (as a proxy for severe impairment) versus fewer than 3 (to be consistent with and to build on existing literature [16–18]). As a secondary outcome, we examined 3 self-reported IADL limitations: preparing own meals, shopping for personal items, and managing money. Because only 3 IADL limitations were examined, we analyzed these as at least 1 limitation versus none.

Our main exposures of interest were self-reported physician-diagnoses of 5 common chronic conditions: arthritis, osteoporosis, hypertension, hypercholesterolemia, and diabetes. Having MCCs was defined as a diagnosis of 2 or more of these conditions and was compared with having no condition or a single condition. In calculating the number of chronic conditions and the yes/no variable for MCCs, variables were constructed to examine only records with valid answers to questions for reporting all 5 chronic conditions.

Three possible confounders — smoking, obesity, and depression — were also included in the analysis. Respondents were categorized as being current smokers if they answered “every day” or “some days” to the question “Do you currently smoke every day, some days, or not at all?,” whereas nonsmokers were defined as those who responded “not at all.” Obesity was defined as having a body mass index of 30 or more, calculated from self-report of height (m^2^) and weight (kg), and depression was defined as ever having received a diagnosis of depression.

Respondents were categorized into 2 age groups: 65 to 74, and 75 or older. Models for the 2 categories of ADL outcomes were examined in 2 age groups to investigate the interaction between MCCs and age. Respondents’ race/ethnicity was categorized exclusively as non-Hispanic white, non-Hispanic black, Asian, or Hispanic. Those whose race/ethnicity was “other” (n = 1) were excluded from all analyses. Income was grouped into 3 categories: very low (<100% of the federal poverty guidelines), low (≥100% to <200% of federal poverty guidelines), and moderate (≥200% of federal poverty guidelines).

### Statistical analysis

Data were weighted by sex, income level, borough, age, and race/ethnicity to be representative of all older NYCHA residents. We used SAS version 9.2 (SAS Institute, Cary, North Carolina) to generate prevalence estimates and 95% confidence intervals (CIs) for BADL limitations, IADL limitations, and chronic conditions by sociodemographic characteristics. Using bivariate analysis, we examined the association of each chronic condition with any BADL limitations in unadjusted models. We used an α level of .05 level to determine significance.

We constructed 2 logistic regression models, with outcomes of at least 1 BADL limitation and 3 to 6 BADL limitations; both models examined the influence of number of conditions on BADL outcomes adjusting for sociodemographic factors. In a separate analysis we added smoking, obesity, and depression to the models, because each of these has a plausible role as a potential confounder. Any IADL limitation was modeled in the same manner as any BADL limitation.

## Results

Our study included 1,016 participants. Most participants were aged 65 to 74, female, and either Hispanic or non-Hispanic black (Table 1). Overall, 28.7% of the sample reported at least 1 BADL limitation. The prevalence of BADL limitations varied by age, income, and sex. Participants aged 75 or older, women, and those with very low income were more likely to have at least 1 BADL limitation or have severe (3–6) limitations than adults younger than 75, men, or those with higher incomes. Asians had a higher point prevalence of BADL limitations than other race/ethnicities, but the CIs were wide. The distribution of age, income, race, and sex was similar across each BADL limitation to the distribution of overall BADL limitations. Differences by sex and by age for IADL limitations were larger than differences for BADL limitations, but they were in the same direction.

**Table 1:** Prevalence of Multiple Chronic Conditions and Basic Activities of Daily Living by Demographic Characteristic, New York City Housing Authority Senior Health Survey, 2009^a^

Characteristic	At Least 1 BADL Limitation		3 to 6 BADL Limitations		At Least 1 Chronic Condition		Multiple Chronic Conditions	
	n	% (95% CI)	n	% (95% CI)	n	% (95% CI)	n	% (95% CI)
**Overall**	283	28.7 (25.6–31.7)	116	12.5 (10.1–14.8)	900	92.9 (91.0–94.8)	771	79.0 (76.0–81.9)
**Age, y**								
65–74	133	23.8 (20.0–27.6)	53	9.4 (6.8–12.1)	512	93.8 (91.4–96.3)	437	79.8 (76.0–83.6)
≥75	150	34.8 (29.8–39.7)	63	16.3 (12.2–20.3)	388	91.7 (88.7–94.8)	334	77.8 (73.3–82.4)
**Sex**								
Male	53	21.7 (16.2–27.3)	18	7.8 (4.2–11.4)	205	85.4 (80.4–90.5)	161	64.3 (57.6–71.1)
Female	230	31.5 (27.8–35.1)	98	14.3 (11.4–17.3)	695	96.0 (94.3–97.6)	610	84.9 (82.1–87.7)
**Race/ethnicity^b^**								
Non-Hispanic black	158	27.8 (24.0–31.5)	57	9.8 (7.3–12.4)	524	94.2 (92.1–96.3)	450	80.5 (77.0–84.0)
Non-Hispanic white	28	28.0 (18.9–37.1)	14	13.5 (6.8–20.3)	80	90.6 (83.4–97.8)	69	77.1 (67.4–86.7)
Asian	19	46.9 (31.2–62.5)	11	27.3 (13.2–41.5)	37	90.1 (80.8–99.5)	31	74.8 (61.0–88.7)
Hispanic	78	27.4 (22.1–32.7)	34	12.9 (8.7–17.0)	258	92.4 (89.0–95.8)	220	78.3 (73.2–83.5)
**Income group^b^^,^ c**								
Very low	170	36.3 (31.6–41.1)	74	17.0 (13.1–20.8)	420	94.0 (91.4–96.6)	364	80.3 (76.1–84.5)
Low	78	24.0 (18.9–29.1)	29	9.2 (5.7–12.7)	288	92.2 (88.6–95.9)	250	79.7 (74.6–84.8)
Moderate	35	17.1 (11.4–22.8)	13	6.5 (2.7–10.2)	191	91.3 (86.9–95.7)	156	74.3 (67.7–81.0)

Abbreviations: BADL, basic activities of daily living; CI, confidence interval.

a Weighted to represent the New York City Housing Authority population by sex, income, borough, age, and race/ethnicity.

b Values for n may not add up to overall N because of missing data.

c Income was grouped into 3 categories: very low (<100% of the federal poverty guidelines), low (≥100% to <200% of federal poverty guidelines), and moderate (≥200% of federal poverty guidelines).

Almost all respondents (92.9%) reported at least 1 diagnosed chronic condition, and a greater proportion of women than men reported at least 1 condition and MCCs. Overall, the most common chronic condition reported was hypertension, and the prevalence was highest (82.7%) among non-Hispanic blacks (Table 2). The distribution of diabetes and osteoporosis also differed by race/ethnicity. The prevalence of diabetes was highest among Hispanics, and the prevalence of osteoporosis was highest among Asians. Hypercholesterolemia and arthritis did not vary significantly by race/ethnicity. For hypercholesterolemia, a greater proportion of those aged 65 to 74 than those aged 75 or older reported being diagnosed, and for arthritis, a greater proportion of those aged 75 or older than those aged 65 to74 reported being diagnosed.

**Table 2:** Prevalence of Chronic Condition, by Demographic Characteristic, New York City Housing Authority Senior Health Survey, 2009^a^^,^ b

Characteristic	Hypertension	Hypercholesterolemia	Osteoporosis	Arthritis	Diabetes
**Overall**	75.7 (72.7–78.7)	58.8 (55.5–62.2)	27.2 (24.2–30.3)	61.3 (58.0–64.6)	37.2 (34.0–40.5)
**Among those with 1 condition**	55.0 (45.5–64.5)	11.8 (5.6–18.1)	2.1 (0.0–4.7)^c^	22.7 (14.6–30.8)	8.4 (3.4–13.4)
**Age, y **					
65–74	73.7 (69.6–77.8)	63.4 (59.1–67.7)	28.8 (24.7–33.0)	58.1 (53.6–62.6)	39.0 (34.6–43.4)
≥75	78.2 (74.0–82.5)	53.0 (47.9–58.2)	25.2 (20.7–29.8)	65.4 (60.5–70.3)	35.0 (30.1–39.9)
**Sex**					
Male	69.4 (63.1–75.7)	49.8 (43.0–56.5)	10.9 (6.6–15.2)	44.0 (37.4–50.6)	35.2 (28.8–41.5)
Female	78.3 (75.0–81.5)	62.5 (58.7–66.3)	33.9 (30.1–37.7)	68.5 (64.9–72.1)	38.0 (34.3–41.8)
**Race/ethnicity**					
Non-Hispanic black	82.7 (79.5–86.0)	56.4 (52.2–60.7)	17.2 (14.1–20.3)	62.5 (58.4–66.6)	36.9 (32.8–40.9)
Non-Hispanic white	66.0 (56.3–75.7)	66.9 (57.0–76.7)	30.0 (20.5–39.5)	58.3 (47.9–68.8)	25.4 (16.4–34.4)
Asian	56.1 (40.6–71.6)	58.0 (42.8–73.3)	56.0 (40.4–71.7)	69.9 (55.8–84.0)	26.0 (12.3–39.7)
Hispanic	73.6 (68.4–78.8)	59.7 (53.8–65.5)	32.7 (27.2–38.2)	59.8 (53.9–65.7)	41.7 (35.8–47.5)
**Income group^d^**					
Very low	76.1 (71.9–80.4)	58.8 (54.0–63.7)	29.9 (25.3–34.4)	64.7 (59.9–69.4)	37.5 (32.8–42.2)
Low	77.2 (72.0–82.3)	60.3 (54.4–66.1)	26.9 (21.5–32.2)	60.9 (55.1–66.8)	35.0 (29.3–40.8)
Moderate	72.4 (65.6–79.2)	56.6 (49.3–63.9)	21.6 (15.3–27.9)	54.2 (46.8–61.5)	40.0 (32.7–47.2)

a Values are percentage (95% confidence interval).

b Weighted to represent the New York City Housing Authority population by sex, income, borough, age, and race/ethnicity.

c Unstable estimates: standard error is 0.062.

d Income was grouped into 3 categories: very low (<100% of the federal poverty guidelines), low (≥100% to <200% of federal poverty guidelines), and moderate (≥200% of federal poverty guidelines).

In modeling the association between having MCCs and having severe impairment (3–6 BADL limitations), the association between the number of chronic conditions and severe impairment was stronger among those aged 75 or older than among those aged 65 to 74 (interaction term *P* < .01 for older age). In separate unadjusted models, each chronic condition was positively associated with having at least 1 BADL limitation. Arthritis showed the strongest association (odds ratio [OR] = 3.4; 95% CI, 2.4–4.9), followed by osteoporosis (OR = 2.5; 95% CI, 1.8–3.5). Cardiovascular conditions had weaker associations with BADL limitations (hypertension, OR = 1.5; 95% CI, 1.1–2.3; hypercholesterolemia, OR = 1.7; 95% CI, 1.3–2.4), as did diabetes (OR = 1.5; 95% CI, 1.1–2.1). Among those with diabetes who reported neuropathy or retinopathy, however, the relationship with at least 1 BADL limitation was relatively strong (OR = 2.7; 95% CI, 1.6–4.5).

Even after adjusting this model for age, sex, income, borough, and race/ethnicity, we observed a strong positive trend between the number of diagnosed conditions and the prevalence of BADL limitations; compared with respondents who had no chronic condition or 1 condition, respondents with 3 conditions were 2.2 (95% CI, 1.3–3.9) times as likely to have at least 1 BADL limitation, those with 4 conditions were 4.3 (95% CI, 2.7–7.6) times as likely, and those with 5 conditions 9.2 (95% CI, 4.3–19.5) times as likely (Table 3). The association between BADL limitations and income also remained strong; those with very low income were 2.5 times as likely to have at least 1 BADL limitation as those with moderate income. These patterns were even more pronounced in the model for severe limitations, although point estimates were less precise.

**Table 3:** Adjusted Odds Ratios (95% Confidence Intervals) of Basic Activities of Daily Living Limitations in Multivariate Model, New York City Housing Authority Senior Health Survey, 2009^a^

Characteristic	Model 1: At Least 1 BADL Limitation		Model 2: 3 to 6 BADL Limitations		Model 1 With Smoking, Obesity, Depression	
	AOR (95% CI)	*P*	AOR (95% CI)	*P*	AOR (95% CI)	*P*
**Age, y **						
65–74	1 [Reference]					
≥75	1.6 (1.1–2.2)	.01	1.9 (1.2–3.1)	.01	1.8 (1.2–2.5)	.005
**Sex**						
Male	1 [Reference]					
Female	1.1 (0.8–1.7)	.51	1.3 (0.7–2.4)	.40	1.0 (0.6–1.5)	.83
**Race/ethnicity**						
Non-Hispanic black	1 [Reference]					
Non-Hispanic white	0.6 (0.4–1.2)	.16	0.8 (0.3–1.9)	.58	0.6 (0.3–1.1)	.10
Asian	2.0 (1.0–3.9)	.05	2.6 (1.1–5.9)	.02	2.1 (0.9–5.0)	.10
Hispanic	0.9 (0.6–1.2)	.44	1.1 (0.6–1.7)	.83	0.8 (0.5–1.2)	.26
**Income group^b^**						
Very low	2.5 (1.5–4.1)	<.001	2.4 (1.2–5.1)	.02	2.6 (1.5–4.4)	<.001
Low	1.3 (0.8–2.3)	.28	1.2 (0.6–2.8)	.61	1.3 (0.7–2.3)	.36
Moderate	1 [Reference]					
**No. of chronic conditions**						
0 or 1	1 [Reference]					
2	1.6 (0.9–2.9)	.12	1.6 (0.6–4.2)	.31	1.3 (0.7–2.5)	.44
3	2.2 (1.3–3.9)	<.001	2.1 (0.9–5.1)	.10	2.1 (1.1–4.0)	.02
4	4.3 (2.5–7.6)	<.001	5.5 (2.4–12.8)	<.001	3.9 (2.1–7.4)	<.001
5	9.2 (4.3–19.5)	<.001	12.2 (4.3–34.3)	<.001	6.0 (2.6–13.9)	<.001
**Other**						
Smokes every day or some days	Not applicable				1.5 (0.9–2.5)	.12
Is obese (BMI ≥30 kg/m^2^)					1.7 (1.1–2.4)	.01
Has ever received a diagnosis of depression					2.0 (1.3–3.2)	<.001

Abbreviations: BADL, basic activities of daily living; AOR, adjusted odds ratio; CI, confidence interval; BMI, body mass index.

a Weighted by sex, income, borough, age, and race/ethnicity to represent the New York City Housing Authority population.

b Income was grouped into 3 categories: very low (<100% of the federal poverty guidelines), low (≥100% to <200% of federal poverty guidelines), and moderate (≥200% of federal poverty guidelines).

After adding diagnosed depression, obesity, and smoking to the model for at least 1 BADL limitation, the dose response by number of chronic conditions was modestly attenuated but remained significant (Table 3). Both obesity (AOR = 1.7; 95% CI, 1.1–2.4) and diagnosed depression (AOR = 2.0; 95% CI, 1.3–3.2) were independently associated with at least 1 BADL limitation; smoking was not.

The model for IADL limitations showed similar patterns to those in the BADL model adjusted for smoking, obesity, and depression — with a few differences. We observed a graded association of IADL limitations with the number of chronic conditions for 3 or more chronic conditions (3 chronic conditions [AOR = 2.5, 95% CI, 1.4–4.5]; 4 chronic conditions [AOR = 3.5, 95% CI, 2.0–6.4]; 5 chronic conditions [AOR = 3.8, 95% CI, 1.7–8.6]). In contrast to the model on BADL limitations, depression (AOR = 2.1, 95% CI, 1.3–3.3) and smoking (AOR = 1.9, 95% CI, 1.2–3.1) were significantly associated, but obesity was not.

The distribution of IADL limitations across chronic conditions was different from the distribution of BADL limitations; in the model for IADL limitations, the ORs for arthritis and osteoporosis were lower than they were in the model for BADLs, but the ORs for hypercholesterolemia, hypertension, and diabetes were similar.

## Discussion

This study demonstrates the heavy burden of MCCs among low-income older adults and establishes a consistent and graded association between the number of chronic conditions and the likelihood of limitations in ADLs (both BADLs and IADLs), despite the heterogeneity of chronic conditions. The prevalence of BADL limitations in this population is almost 5 times greater than the 2007 overall national estimate of 6% for the same age group (19). The strength of the association of MCCs with BADLs was more than additive as the number of chronic conditions increased; it was also greater than the association of any 1 condition with BADLs. Many studies have documented the negative health impact and high cost of MCCs; findings from this study confirm the importance of developing and testing tailored interventions for managing MCCs and preventing additional conditions to reduce the potential for BADL limitations among older, lower-income adults in community settings.

Findings from a systematic review of studies on multimorbidity suggest the burden or impact of MCCs often varies because of different operational definitions of multimorbidity (20); the term has been defined as encompassing as few 4 conditions and as many as 22. Our study included 5 common chronic conditions that are easily screened for and are treated for the most part by nonspecialty primary care physicians; we included these 5 conditions to characterize their influence on functionality and provide practical information to primary care and public health practitioners. We included depression as a potential confounder rather than as a chronic condition; because our measurement was of ever having received a diagnosis of depression, we could not validate it to be a condition at the time of the survey. We found that obesity and a diagnosis of depression were independently associated with BADL limitations. This finding is consistent with other studies’ findings of greater limitations among those who are depressed (21,22) or obese (23). Both depression and obesity may serve as barriers to disease self-management (24), although depression may also be a marker for chronic disease duration or severity (25) or associated with BADLs for some other reason.

Other research on patterns of chronic conditions supports the importance of recognizing MCCs, emphasizing interventions that focus on clusters of disease instead of a single disease to improve efficacy and cost efficiency. A recent review of randomized controlled trials on the efficacy of interventions in patients with multimorbidity in primary care and community settings stressed the importance of defining a set of generic health outcomes that can be used for different combinations of disease and compared across studies (26). One study showed BADL limitations to have these outcome characteristics (ability to be used for different combinations and compared across studies), and BADL limitations are strongly associated with a range of poor health and quality-of-life outcomes (12). Indeed, an increase in the number of BADL limitations may both reflect and exacerbate a downward health spiral in older age. The use of BADLs as health outcomes may allow for more focused decision making on patient care to achieve the greatest overall effect on health across a range of condition constellations.

Although the prevalence of MCCs in our study population mirrors that of the United States as a whole, we identified disproportionately high levels of BADL limitations in our racially/ethnically diverse population. For low-income populations, the greater burden of BADL limitations may be related to the greater proportion of older adults who have 3 or more chronic conditions. In our adjusted models, the number of chronic conditions — especially having 4 or 5 chronic conditions — was the strongest predictor of having at least 1 BADL limitation. BADL limitations were also negatively associated with income level; lifelong lack of access to care, elevated environmental stress, or other issues among low-income populations may worsen the effects of chronic conditions on BADL limitations (27). 

Cost-related issues are of special concern in a resource-limited community. Our sample evidenced some disease clustering and a higher risk of MCCs with increasing age, and although our data are cross-sectional, we may infer that chronic conditions accrue over time. This inference makes prevention especially relevant. Our findings also suggest that screening for and treatment of MCCs should include an assessment of ADL limitations, particularly in a low-income population, who are more likely to have limitations and for whom the difficulty of successful aging in place may be greater. The association of specific diseases with ADL limitations suggests that disease clustering may help guide screening and prevention measures.

This survey provided an opportunity to understand a range of health issues affecting older low-income adults in a behavioral and social context. Our population was uniform in characteristics potentially important to disability: all respondents initially qualified for public housing on the basis of income, and eligibility for Medicare was nearly universal.

Our study has several limitations. Because the sample was cross-sectional, we were not able to assess temporal order of disease accrual, identify the onset or duration of any chronic condition or ADL limitation, or evaluate disease progression. That said, our findings support earlier research that showed co-occurrence of disease may be an important determinant of loss of capacity to perform BADLs (12). Only limited clinical data were available. Diabetes types 1 and 2 were not differentiated in this survey, and osteoarthritis was not differentiated from rheumatoid arthritis. Generalizability is limited to similar populations — low-income and racial/ethnic minority populations have special challenges in access to care and health literacy that in turn may influence diagnosis, disease management, and subsequent health outcomes (28). IADLs and BADLs are sometimes combined into 1 scale to mark the progress of disability (12,29). In our study, we had only 3 IADL items, limiting the comparison with other studies and limiting the validation of combined scales in other research (30).

In this diverse, low-income, community-based sample of older adults, where chronic conditions are widespread, the number of chronic diseases has a relatively strong association with BADL performance. This association suggests that diagnosis of an initial disease may mark an important time to initiate preventive measures for the onset of other conditions to limit the most severe health effects and delay functional loss. Knowledge of disease clustering may inform prevention efforts for both disease and limitation accrual, and screening for smoking, obesity, and depression may help identify those at higher risk for BADL limitations.
